# Efficient Plantlet Regeneration from Branches in *Mangifera indica* L.

**DOI:** 10.3390/plants13182595

**Published:** 2024-09-17

**Authors:** Huijing Zhou, Jinglang Sun, Keyuan Zheng, Xinyuan Zhang, Yuan Yao, Mulan Zhu

**Affiliations:** 1 Shanghai Key Laboratory of Plant Functional Genomics and Resources, Shanghai Chenshan Botanical Garden, Shanghai 201602, China; zhouhuijing9527@163.com (H.Z.); 18846826439@163.com (J.S.); kyzheng@cemps.ac.cn (K.Z.); 2 National Key Laboratory of Plant Molecular Genetics (NKLPMG), Chinese Academy of Sciences (CAS) Center for Excellence in Molecular Plant Sciences, Shanghai 201602, China; 3 College of Forestry, Beijing Forestry University, Beijing 100107, China; 18611979657@163.com; 4 National Key Laboratory for Tropical Crop Breeding, Sanya Research Institute, Institute of Tropical Bioscience and Biotechnology, Chinese Academy of Tropical Agricultural Sciences, Haikou 570102, China; yaoyuan@itbb.org.cn

**Keywords:** Mango, in vitro regeneration, organogenesis, adventitious shoot induction, genetic fidelity

## Abstract

Mango (*Mangifera indica* L.) is one of the most significant tropical and subtropical fruit species, with high ecological and economic value. However, research on the in vitro culture of mangoes is relatively weak, so establishing an efficient and stable mango plant regeneration system is of great significance. In this study, a preliminary mango regeneration system was established with *Mangifera indica* L. cv. Keitt from young branches as the starting explants. The results showed that the optimal plant growth regulator (PGR) formula for direct adventitious shoot induction on the branches was 1 mg/L 6-benzylaminopurine (6-BA) + 0.1 mg/L a-naphthaleneacetic acid (NAA), with an adventitious shoot induction rate of 73.63% and an average of 6.76 adventitious shoots. The optimal basal medium for adventitious shoot induction was wood plant medium (WPM), with an adventitious shoot induction rate of 63.87% and an average of 5.21 adventitious shoots. The optimal culture medium for adventitious shoot elongation was WPM + 1 mg/L 6-BA + 0.5 mg/L NAA, with an adventitious shoot elongation rate of 89.33% and an average length of 5.17 cm. The optimal formula for the induction of mango rooting was Douglas fir cotyledon revised medium (DCR) + 3 mg/L indole-3-butyric acid (IBA), with a maximum rooting rate of 66.13% and an average rooting quantity of 6.43. The genetic fidelity of the in vitro-regenerated plants was evaluated using inter-simple sequence repeat (ISSR) molecular markers. There was no difference between the in vitro-regenerated plants and the parent plant. This study provides an efficient and stable propagation system for *Mangifera indica* L., laying the foundation for its rapid propagation and genetic improvement.

## 1. Introduction

Mango (*Mangifera indica* L.), a fruit species and evergreen tree, is a member of the Anacardiaceae family. It originated in India and Malaysia and is now widely distributed in subtropical and tropical regions [1]. Mango is also one of the top five fruits in the world, known as the “King of Tropical Fruits” [1], and is one of the main cash crops in its growing areas. In view of the ongoing rise in mango outputs worldwide, the rapid and efficient production of high-quality adventitious shoots has become an important topic in mango research [2,3]. There are still many problems in the production of mango adventitious shoots. Due to the fact that mangoes are highly cross-pollinated species, the offspring selected through sexual reproduction have unstable traits [4,5]. Grafting is beneficial in maintaining the excellent characteristics of mango varieties and unifying the phenological period of the orchard. However, currently, some rootstocks die from cold damage or suffer from serious gum disease and root rot during the high yield period [6,7,8,9]. Cutting propagation has the problem of difficult rooting, and the propagation coefficient of high-pressure propagation is low [10,11,12]. Research on mango tissue culture began as early as the 1980s. American scholar Litz R.E. obtained somatic embryos through the tissue culture of mango ovules [13]. In 1989, Dewald et al. used the nucellus tissue of Paeeis and James Saigon as explants, induced somatic cell embryos in improved Gamborg’s B-5 Medium (B5), and continued to perform culturing to obtain complete plants [14]. In 1995, Raghuvanshi et al. reported the induction of callus tissue from the tender leaves of mangoes and successfully obtained complete plants [15]. Wu Yongjie used the cotyledons and ovules of mangoes as explants and induced embryogenic cultures on MB medium. Mature cotyledons were formed through somatic embryogenesis, and new plants were finally obtained [16]. Xiao Jiening used mango top shoots, petioles, flower branches, young fruits, cotyledon nodes, root tips, and leaves as explants for tissue culture [17]. After 7 days of germination, three to seven axillary shoots were induced from the cotyledon nodes of decapitated young adventitious shoots. After subculture, the roots were induced on rooting medium for 1 week before taking root [17]. Conde conducted regeneration studies on four types of mango: ‘Ataulfo’, ‘Sabre’, ‘Gomera-4’, ‘Irwin’, and ‘Keitt’. Among them, ‘Keitt’ had the best regeneration effect, with a maximum regeneration rate of 63.15%, and approximately four shoots were obtained from each regenerated explant [18]. Despite the increasing research on mango tissue culture both domestically and internationally, the necrosis of explants, the browning of the culture media due to phenolic material release, endogenous contamination, sluggish development, and challenges with root induction in mango tissue culture are still important issues [19,20]. At present, mangoes still lack a stable in vitro regeneration system. Therefore, the development of effective in vitro culture systems to ensure clean cultures and optimization of media formulation and growth conditions for multiple shoot production are essential for further genetic resource conservation and breeding efforts [21].

The goal of this study is to create a reliable in vitro regeneration system for *Mangifera indica* L., increase the multiplication and rooting rates, shorten the cultivation cycles, expand the propagation coefficients, and continuously screen and optimize the cultivation conditions, in order to provide a technical reference for the genetic improvement, germplasm resource preservation, and innovation of mangoes.

## 2. Results

### 2.1. Adventitious Shoot Induction from Branches of Mangifera indica L.

**Figure 1:**
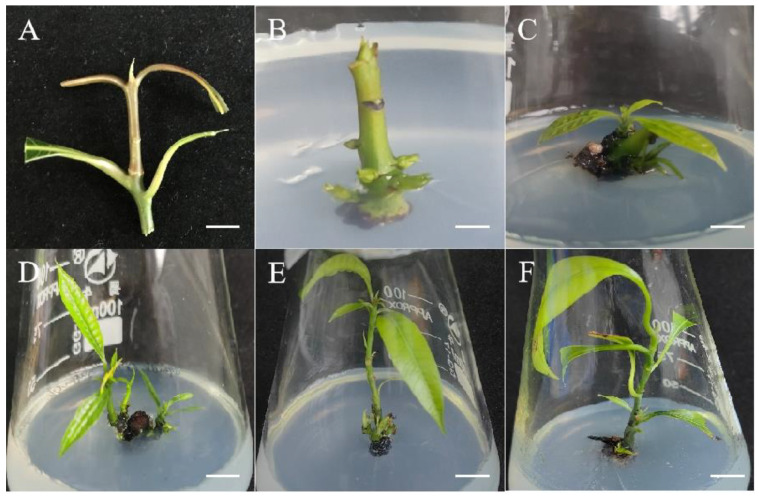
In vitro shoot induction and development of *Mangifera indica* L. cv. Keitt. from young branches. (**A**): Young branches of mango, bar = 1 cm. (**B**): Adventitious shoot induction, culture for 29 days, bar = 1 cm. (**C**–**E**): Elongated new plantlets. (**C**) Culture for 40 days; (**D**) culture for 66 days; (**E**) culture for 95 days, bar = 2 cm. (**F**): Initiation of roots, culture for 106 days, bar = 2 cm.

Using young branches of mango as the starting explant, we established a preliminary mango regeneration system (Figure 1). The aseptic materials could be effectively obtained through treatment with 75% ethanol for 1 min and PB (plant preservative mixture and 1% benzyldodecyldimethylammonium bromide mixture) for 4 min. The adventitious shoots were generated during approximately four weeks of culture on wood plant medium (WPM) + 0.7 mg/L 6-benzylaminopurine (6-BA) + 0.07 mg/L a-naphthaleneacetic acid (NAA) (Figure 1B), and the adventitious shoots were gradually elongated on WPM + 0.1 mg/L 6-BA + 0.01 mg/L NAA (Figure 1C–E). After three passages, the degree of lignification of the mango plantlets increased. We used sterile mango plantlets in this growth state as the starting explants for the subsequent regeneration experiments (Figure 1F). Compared with traditional methods, this approach can be used to prepare mango regeneration explants without seasonal limitations.

### 2.2. Effect of PGRs on Adventitious Shoot Induction

**Figure 2:**
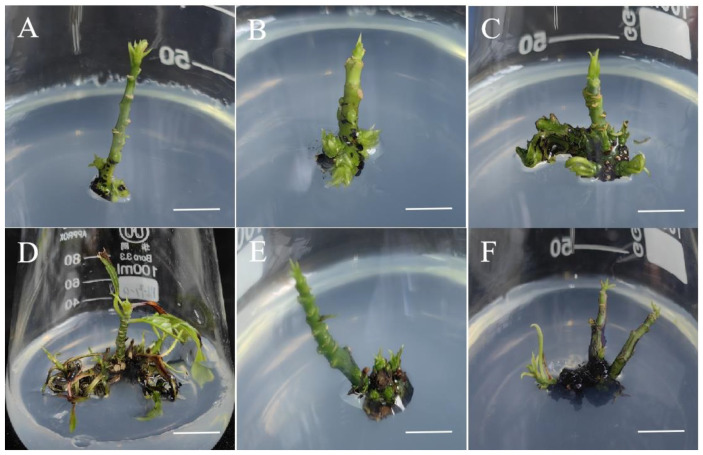
The effect of PGRs on the adventitious shoot induction of *Mangifera indica* L. cv. Keitt. (**A**): Combination of 0.5 mg/L 6-BA + 0.05 mg/L NAA, cultivated for 24 days, bar = 0.8 cm. (**B**): Combination of 0.5 mg/L 6-BA + 0.1 mg/L NAA, cultivated for 24 days, bar = 1 cm. (**C**): Combination of 1 mg/L 6-BA + 0.1 mg/L NAA, cultivated for 25 days, bar = 0.8 cm. (**D**): Combination of 1 mg/L 6-BA + 0.2 mg/L NAA, cultivated for 42 days, bar = 1 cm. (**E**): Combination of 2 mg/L 6-BA + 0.2 mg/L NAA, cultivated for 26 days, bar = 1.2 cm. (**F**): Combination of 2 mg/L 6-BA + 0.4 mg/L NAA, cultivated for 28 days, bar = 1.5 cm.

Plant growth regulators (PGRs) play an important role in the process of in vitro plant regeneration, and different types and concentrations of PGRs affect the growth and development of plant tissue and organs. This study investigated the effects of 6-BA and NAA on direct adventitious shoot induction in mangoes. There were significant differences in the induction of adventitious shoots in WPM with various combinations of growth regulators added (Table 1). When the concentration range of 6-BA was 0.5–1 mg/L and the concentration of NAA was 0.05–0.2 mg/L, the induction rate and number of mango adventitious shoots increased with the increase in the 6-BA and NAA concentrations (Table 1), and the adventitious shoots appeared to be in a healthy state (Figure 2A–C). When the cytokinin 6-BA/auxin NAA concentration ratio was 10/1, the effect of mango adventitious shoot induction was better (Table 1, Figure 2C,E). In the WPM with 0.1 mg/L NAA and 1 mg/L 6-BA added, the direct adventitious shoot induction rate of the branches of *Mangifera indica* L. cv. Keitt was the highest, reaching 73.63%; moreover, the average number of adventitious shoots was 6.76, and the plant exhibited good growth conditions, with thick green and stretched leaves (Figure 2C). The attainment of a specific biological response necessitates the achievement of a precise concentration threshold, irrespective of the ratio of cytokinin 6-BA to auxin NAA being 10:1 or lower. There were relatively few adventitious branches and a very low induction rate in the culture medium containing 0.5 mg/L 6-BA and 0.05 mg/L NAA (Table 1, Figure 2A). However, when the concentrations of 6-BA and NAA were too high, this had a negative effect on the induction of adventitious shoots. When the concentration of 6-BA was 2 mg/L and the concentration of NAA was 0.2 mg/L, the induction rate of the mango adventitious shoots was 62.07%, with an average of 4.73 adventitious shoots (Table 1), and dead shoots appeared on the 42nd day of cultivation (Figure 2D). In the medium supplemented with 2 mg/L of 6-BA and 0.4 mg/L of NAA, the aboveground regeneration rate was 50.85% and the number of shoots per explant was 1.20 (Table 1, Figure 2F). Therefore, the shoot regeneration rate and number of adventitious shoots induced by different formulas are inconsistent and disproportionate (Table 1).

### 2.3. Effect of Basic Culture Medium on Induction of Adventitious Shoots

**Figure 3:**
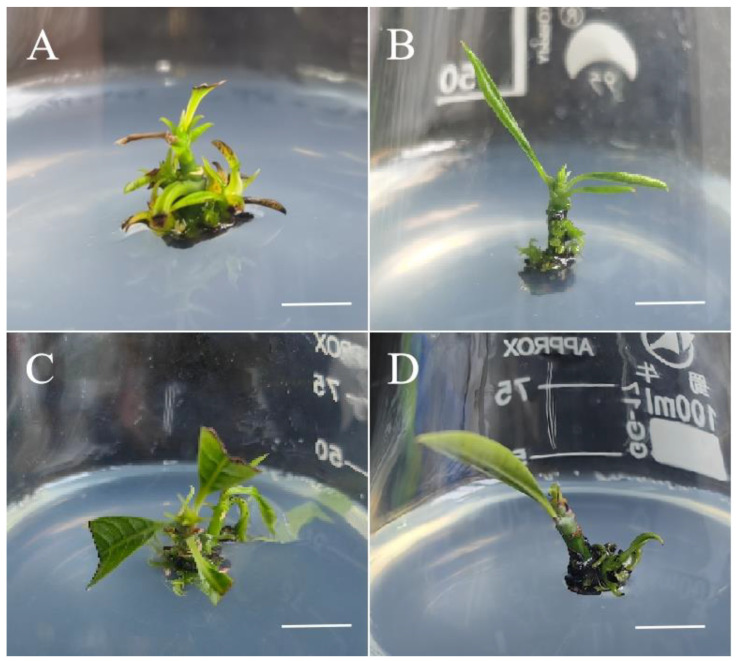
The effect of the basal medium on the adventitious shoot induction of *Mangifera indica* L. cv. Keitt. (**A**): Regenerated plantlets in MS medium, cultivated for 35 days, bar = 2 cm. (**B**): Regenerated plantlets in 1/2 MS medium, cultivated for 28 days, bar = 2 cm. (**C**): Regenerated plantlets in WPM, cultivated for 47 days, bar = 2 cm. (**D**): Regenerated plantlets in DCR medium, cultivated for 37 days, bar = 2 cm.

We transferred sterile materials to different basic media for adventitious shoot induction, with 1 mg/L 6-BA and 0.1 mg/L NAA added to each medium. As shown in Table 2 and Figure 3, the best induction effect was achieved with the WPM basic medium, with an induction rate of 63.87% and an average of 5.21 adventitious shoots (Figure 3C). This was followed by the Murashige and Skoog (MS) and 1/2 MS media, with induction rates of adventitious shoots of 53.72% and 56.70%, respectively (Figure 3A), and with an average of 3.56 and 4.83 adventitious shoots, respectively (Figure 3B). In the Douglas fir cotyledon revised (DCR) medium, the induction rate and number of mango adventitious shoots were both very low, with an induction rate of only 47.92% and only 2.15 adventitious shoots, indicating a poor induction effect (Figure 3D).

### 2.4. Effects of 6-BA on Elongation and Growth Status

**Figure 4:**
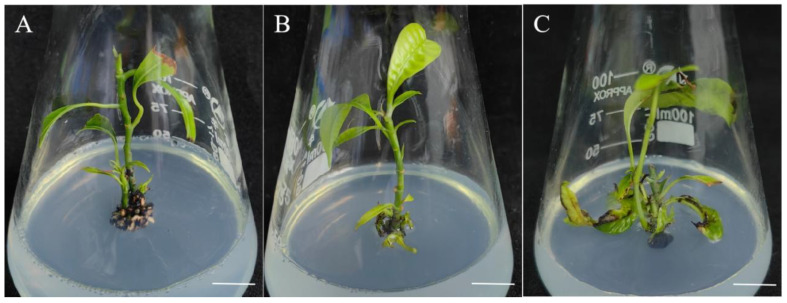
Effects of 6-BA on *Mangifera indica* L. cv. Keitt elongation and growth conditions. (**A**): Regenerated plantlets with 0.1 mg/L 6-BA, cultivated for 60 days, bar = 1 cm. (**B**): Regenerated plantlets with 0.5 mg/L 6-BA, cultivated for 60 days, bar = 1 cm. (**C**): Regenerated plantlets with 1.0 mg/L 6-BA, cultivated for 60 days, bar = 1 cm.

In order to induce elongation, the adventitious shoots were moved to WPM containing varying concentrations of 6-BA. As shown in Table 3 and Figure 4, 6-BA significantly affected the elongation of *Mangifera indica* L. When the concentration of 6-BA was 0.1 mg/L, the elongation of the mango adventitious shoots reached 75%, the average height of the adventitious shoots was 4.51 cm (Table 3), and the leaves were healthy and green (Figure 4A). The optimal induction effect, with an adventitious shoot elongation rate of 89.33% and a maximum average stem length of 5.17 cm, was obtained at a concentration of 0.5 mg/L of 6-BA (Table 3, Figure 4B). With an increase in the 6-BA concentration, the adventitious shoot elongation rate exhibited a tendency of initially rising and then declining. When the amount of 6-BA was 1.0 mg/L, the elongation rate and length of the adventitious shoots were lower than those at the other two concentrations of 6-BA, and the edges of the bottom leaves were charred (Table 3, Figure 4C).

### 2.5. Effect of IBA on Rooting of Mangifera indica L.

**Figure 5:**
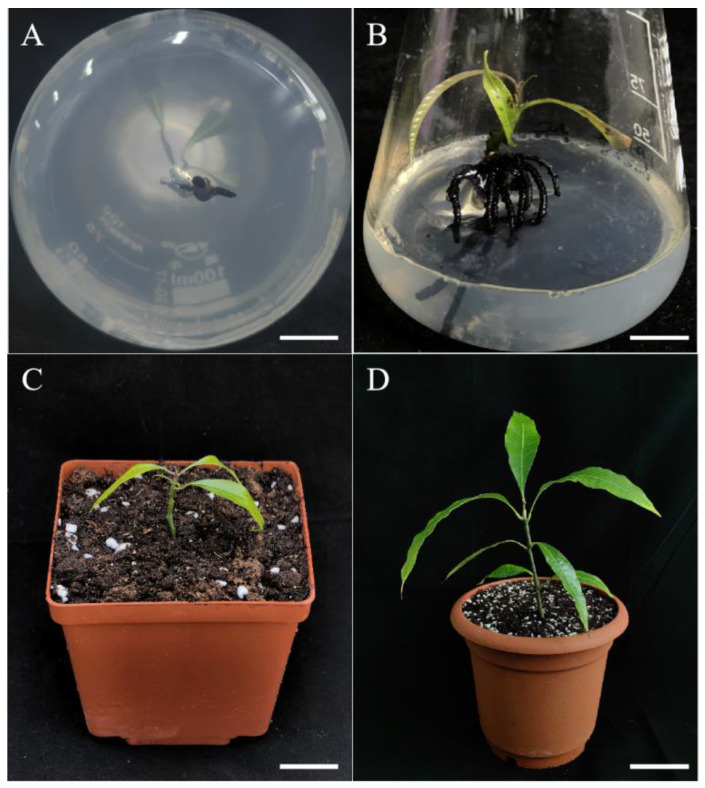
Rooting and acclimatization of *Mangifera indica* L. cv. Keitt regenerated shoots. (**A**): Shoot regeneration culture for 32 days, bar = 1 cm. (**B**): Shoot regeneration culture for 2 months, bar = 1 cm. (**C**): Plants acclimatized for 2 weeks, bar = 3 cm. (**D**): Plants acclimatized for 8 months, bar = 10 cm.

**Figure 6:**
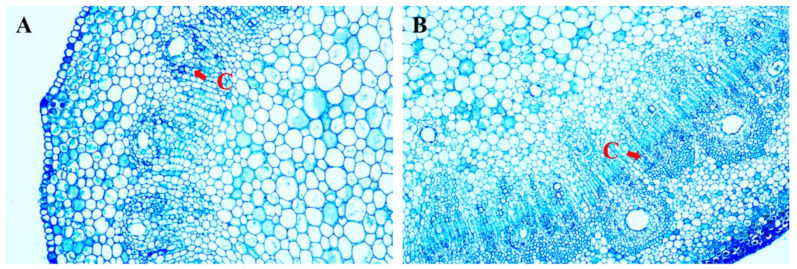
Cross-sectional structure of mango stem internodes. (**A**): Cross-sections of CK (culture in DCR medium without IBA). (**B**): Cross-sections treated with 3 mg/L IBA. C, Cambium.

Indole-3-butyric acid (IBA) and NAA are the primary plant growth regulators utilized in plant root cultures. In this study, IBA was used as the rooting-inducing hormone for mangoes. As shown in Table 4, in the DCR medium, the mango rooting number and rooting rate exhibited a tendency of initially increasing and then dropping with an increase in the IBA concentration. When the IBA content was 3 mg/L, rooting began after approximately 4 weeks of cultivation (Figure 5A). Moreover, the average rooting number of adventitious shoots in mango was the highest, reaching 6.43, and the rooting number was also the best among all treatments, with a rooting rate of 66.13%. The mango’s adventitious root induction was poor at concentrations of IBA of 1 mg/L and 4 mg/L, which led to a low rooting rate, fewer adventitious roots, and delayed growth. In the medium with 3 mg/L of IBA added, the activity of the cambium cells in the mango stem segment was high (Figure 6B), and the width of the cambium area and the number of cell layers were significantly larger than those in the control group (Figure 6A). The above results show that IBA promoted the division of the cambial cells. Therefore, the DCR medium with 3 mg/L IBA added was the most suitable medium for *Mangifera indica* L. cv. Keitt rooting.

### 2.6. Acclimatization

Two weeks after transplanting the plants, the lid was removed. Approximately 4–6 weeks after transplanting them, the plantlets successfully adapted to the soil and external environment, with a survival rate of 42.67%. After 8 months, the transplanted mango plantlets had a relatively healthy leaf-like structure, with dark green leaves that exhibited a normal plant morphology (Figure 5D).

### 2.7. Genetic Fidelity Assessment

In this study, a total of 20 ISSR primers were used to evaluate the genetic fidelity of the mangoes. Among them, eight ISSR primers were used to generate 48 different monomorphic bands, which were used for subsequent PCR amplification reactions (Table 5). With banding patterns ranging from four (UBC808 and BUC848) to nine (UBC836), an ISSR primer produced an average of six bands for the ISSR markers (Table 5).

**Figure 7:**
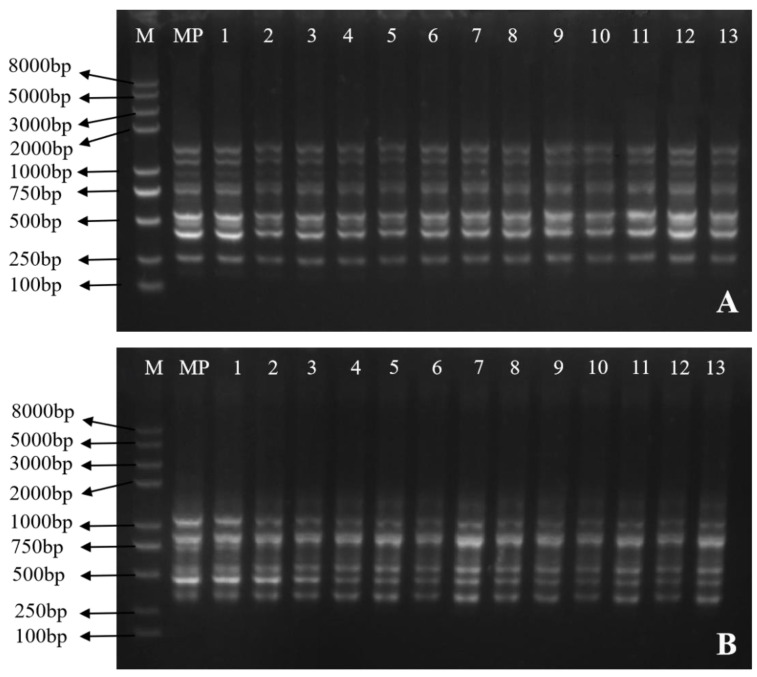
Genetic fidelity of regenerated plants, determined using ISSR molecular markers. Profiles were obtained from primers UBC836 (**A**) and UBC888 (**B**). Lane M: molecular marker (100 bp–8 kb). MP: mother plant. Lanes 1–13: regenerated plants.

According to Table 5, the bands’ sizes varied from 250 to 2000 bp. Figure 7 displays two of the ISSR profiles. Nine bands ranging in size from 250 to 2000 bp were produced by the UBC836 primer (Figure 7A), while six bands ranging in size from 300 to 1500 bp were produced by the UBC888 primer (Figure 7B). The findings show that there was no somaclonal variation between the mother plant of *Mangifera indica* L. cv. Keitt and the in vitro-regenerated plants. 

## 3. Discussion

For the initiation of explants in mango tissue culture, various parts, such as the ovules, nucellus, ex vivo hypocotyls, stem tips, young leaves, stem segments, and anthers, are commonly used [18,20,22]. These are then disinfected and inoculated onto the appropriate culture medium for callus or adventitious shoot induction. This study established an effective mango adventitious shoot regeneration system using young branches from seven-year-old mango trees as starting explants, and experiments were conducted on adventitious shoot induction, elongation, and root induction using sterile adventitious shoots as the starting explants. This method can effectively avoid material contamination and improve the regeneration efficiency. Moreover, it is not limited by the season and plants can be produced year-round.

Common plant growth regulators include 6-BA, 2,4-dichlorophenoxyacetic acid (2,4-D), NAA, etc. Specifically, cytokinins, exemplified by 6-BA, exhibit a stimulatory effect on the induction and differentiation of plant organs; conversely, auxins, such as 2,4-D, play a pivotal role in promoting the proliferation and growth of plant callus tissue [23]. B. L. Lad et al. found, in their study on Carabao mangoes, that the largest number of heart-shaped embryos was obtained when the concentrations of 6-BA and Kinetin (KT) in the culture medium were 4.44 µmol/L and 4.65 µmol/L, respectively [24]. The findings of Muhammad’s study on the regeneration of mango stem tips revealed that the induction rate of adventitious shoots was 55.56% at a concentration of 1 mg/L of 6-BA and 83.30% at a concentration of 3 mg/L of 6-BA [25]. A number of researchers have suggested that elevated levels of cytokinin in the culture media might cause the polyphenol oxidase activity to rise in the callus tissue, which would brown the tissue and seriously damage the explants and callus tissue [26,27]. This experimental investigation demonstrates that, within a specific dosage range, 6-BA promotes the induction of mango adventitious shoots. When the content of 6-BA was 1 mg/L, the induction rate of the mango adventitious shoots reached 73.63%. When the 6-BA concentration reached 2 mg/L, the proliferation of the mango adventitious shoots was inhibited, which is consistent with previous studies. In the process of mango tissue culture, auxin is also needed. The main types of auxin are 2,4-D, NAA, IBA, and auxin (IAA), with a concentration of 5.4 µmol/L of NAA being commonly used [20]_._ Previous studies have shown that a lower content of 2,4-D is beneficial for the formation of the mango callus, while more potent concentrations of 2,4-D are not conducive to the proliferation and differentiation of the callus [28,29]. The ratio of cytokinin to auxin can also influence the adventitious shoot incidence during in vitro plant regeneration. Research on the regeneration of Populus davidiana showed that the optimal efficiency of adventitious shoot regeneration was achieved when the ratio of 6-BA to NAA was 10/1 [30]. Wu Jiayi et al.‘s study on the stem regeneration of Eucommia ulmoides showed that the optimal hormone ratio for the induction of axillary shoot germination to form clustered adventitious shoots was 6-BA/NAA = 5/1 [31]. There may be notable variations across various plants and even distinct tissue organs, as the precise amounts and concentrations of these plant growth regulators have not been fully established in different plants. According to our study’s results, mangoes regenerate most effectively when the 6-BA/NAA ratio is 10/1. The concentrations of PGRs are a crucial factor influencing their efficacy in modulating plant growth and development. In this study, the proliferation rate of the mango adventitious shoots showed a trend of first increasing and then decreasing with the increase in the plant growth regulator concentration. This phenomenon has also been documented in the process of other species’ in vitro regeneration [32], indicating that the proportions and concentrations of PGRs are important for in vitro plant regeneration.

Different mango tissue types exhibit varying adaptability to culture media, with immature embryos commonly cultivated using modified B5 medium [33,34,35,36]; in contrast, young mango leaves are typically cultured using MS medium [15]. According to Wang Xiaofeng’s research, mango hypocotyls cultured in vitro can thrive in four basic media, namely, Driver and Kuniyuki and McGranahan et al. medium (DK), MS, 1/2 MS, and WPM, with normal growth occurring exclusively with DK and WPM media [37]. In this study, the WPM showed the best regeneration effect on *Mangifera indica* L. cv. Keitt. In 1980, Lloyd and McCown created WPM specifically for the stem tip cultivation of Osmanthus altissima [38]. It is characterized as a low-mineral medium and is devoid of ammonium nitrogen. This demonstrates the crucial role of specific nutrients in the developmental processes of woody plants. This knowledge is essential in tailoring the basic culture medium formula to cater to the unique physiological requirements of woody plants.

In mango tissue culture, rooting culture has always been a significant difficulty. Studies have shown that there are two main means of adventitious root formation: In the first, the columella cells and columella-like cells in the plant cortex develop into a callus and then transform into adventitious roots, which is called indirect de novo root regeneration (DNRR). In the second, the cambium cells adjacent to the vascular bundle in the plant directly develop into adventitious roots, which is called direct DNRR [39,40]. In general, in the process of mango tissue culture, direct DNRR is the main strategy. In recent years, through the study of the Arabidopsis leaf rooting system, the direct DNRR process has been divided into three consecutive stages at the molecular level: early signaling, auxin accumulation, and cell fate transition. The process and mechanism of direct DNRR in woody plants are similar to those in Arabidopsis [41,42]. Auxin is a key hormone in the DNRR process and is important for adventitious root induction in woody plants. Ara et al. treated mangoes with a 5.0 mg/L IBA pulse for 24 h and achieved good rooting effects on an auxin-free culture medium. The experimental results showed that IBA had good effects on rooting and root growth [28]. Lilian F. et al. used Ma15 (Gamborg B5 Medium macronutrients, MS micronutrients, MS vitamins, and Fe-EDTA) medium supplemented with 2% sucrose, 200 mL/L coconut water, and 50 mg/L activated carbon to induce mango rooting [43]. This study referred to previous research and selected DCR as the basic medium to induce mango rooting through IBA. The rooting effect was good in DCR medium supplemented with 3 mg/L IBA, with a high rooting quantity and rate, making it a relatively efficient mango rooting induction system.

Maintaining the genetic integrity of a species is one of the most important conditions in determining the success of in vitro reproduction. Traditional methods for the detection of genetic variation in varieties mainly rely on the plant morphology for identification and analysis. However, due to the fact that many genetic variations in plants may not necessarily indicate traits, it is difficult to accurately detect them solely through morphological observation. Molecular marker technology can reflect changes in base sequence fragments at the DNA level, thus elucidating the genetic stability of tissue-cultured adventitious shoots at the molecular level. Recently, ISSRs have been extensively utilized to examine the genetic stability of regenerated woody plant species, such as *Simmondsia chinensis* [44], *Sapium sebiferum* Roxb [45], *Albizia lebbeck*, and *Morus alba* L. cv. Chinese white [46,47]. Our study investigated the genetic fidelity between the regenerated plants and mother plant through ISSR analysis, and the regenerated plants were confirmed to be “true to type”, with the ISSR bands of the mother plant and the regenerated plants being comparable.

Although the studied system effectively induced the in vitro regeneration of branches, browning still inevitably occurs in mango tissue during the cultivation process. Inhibiting browning is crucial in tissue culture and transgenic research on mangoes. In future research, more effective formulas to curb browning will be explored. The induction of mango rooting has always been a difficulty in mango tissue culture research. Although this study successfully induced mango rooting and the number of roots was large, there are still problems associated with the low survival rate of transplanting. In the future, we will use this regeneration system to explore a comprehensive large-scale breeding system and conduct in-depth research on the growth mechanism of mangoes. We hope that this regeneration system will promote the development of mango germplasm protection and breeding work.

## 4. Materials and Methods

### 4.1. Plant Materials and Culture Conditions

We used the young branches of mango trees grown in a greenhouse for seven years as the starting explants, and the materials were taken from Shanghai Chenshan Botanical Garden. To ensure optimal growth conditions, the cultivation medium was adjusted to a pH of 5.8, and the explants were maintained at a controlled temperature of 25 ± 2 °C. The cultivation environment was further supplemented with a light intensity of 33.6 mol/m^2^/s and a photoperiod regimen of 16 h of light alternating with 8 h of darkness.

### 4.2. Preparation of Sterile Materials

We selected healthy young mango branches and cut them into 3–5 cm sections (retaining 1–2 nodes). After rinsing the branches (Figure 1A) under tap water for 0.5 h, they were sterilized in 75% alcohol for 1 min and PB for 4 min, and they were inoculated into WPM + 0.7 mg/L 6-BA + 0.07 mg/L NAA and cultured for 2 weeks to obtain sterile material. Cultivation was continued on WPM + 0.7 mg/L 6-BA + 0.07 mg/L NAA medium to induce the occurrence of adventitious shoots (Figure 1B). We transferred the adventitious shoots to WPM + 0.1 mg/L 6-BA + 0.01 mg/L NAA medium for elongation induction. They were transferred every 4 weeks and subcultured 2–3 times to improve the degree of lignification in the mango plantlets (Figure 1F). We added 30 g/L sucrose and 5 g/L agar to the basic medium and sterilized it with high-pressure steam at 0.105 MPa pressure and 121 °C for 20 min. All components of the culture media and sucrose and agar used in this study were purchased from the Hangzhou Lin’an Bottled Science Experiment Supplies Business Department (Hangzhou, China). All PGRs were purchased from Hangzhou Morebetter Biotechnology Co., Ltd. (Hangzhou, China). The plant preservative mixture was purchased from Yeasen Biotechnology Co., Ltd. (Shanghai, China).

### 4.3. Induction of Adventitious Shoots from Branches 

Septic plantlets with a high lignification degree were subcultured 3 times on WPM + 0.1 mg/L 6-BA + 0.01 mg/L NAA medium (Figure 1F) as the starting explants for adventitious shoot induction. To investigate the effects of PGRs on mango adventitious shoot induction, the sterile materials were transferred to WPM supplemented with 0.5–2 mg/L 6-BA and 0.05–0.4 mg/L NAA for the direct induction of adventitious shoots. Additionally, to evaluate the impact of basic media on mango adventitious shoot induction, the sterile materials were inoculated onto MS [48], half-strength (1/2) MS, WPM [38], and DCR [49] media, each supplemented with 1 mg/L 6-BA and 0.1 mg/L NAA. Each treatment involved inoculating 20 explants, with 1 explant per container (100 mL triangular glass bottle), and three repetitions of each treatment were conducted.

### 4.4. Elongation of Adventitious Shoots

When the adventitious shoots had grown to a length exceeding 1 cm, we transferred them to WPM and each one was specifically supplemented with varying doses of 6-BA: 0.1 mg/L, 0.5 mg/L, or 1.0 mg/L. This transfer aimed to further promote the elongation of the in vitro shoots under the influence of different 6-BA concentrations. Each culture medium was combined with 5 g/L agar and 30 g/L sucrose at a pH of 5.8. Twenty explants were used for inoculation in each treatment, with one explant per bottle (100 mL triangular glass bottle), and each treatment was repeated 3 times. They were transferred once after 4 weeks; after 8 weeks, we determined the average length of shoot elongation and the adventitious shoot elongation rate.

### 4.5. Induction of Roots and Acclimatization

We transferred sterile adventitious shoots with a good growth status and high degree of lignification to rooting induction medium, selected the DCR medium as the basic medium, and added 1, 2, 3, or 4 mg/L IBA to the medium. Each culture medium was combined with 5 g/L agar and 30 g/L sucrose at a pH of 5.8. We inoculated 20 explants per treatment, with one explant per bottle (100 mL triangular glass bottle), and repeated each treatment 3 times. They were transferred once every 4 weeks and we measured the number and rooting rate of the roots. For acclimatization, we chose regenerated plants with a large number of roots and strong development. We opened the bottle cap, added the recommended amount of tap water, and kept the plantlet in a cool place for 3 days. After this, we removed the plantlets and subjected them to quick washing under a faucet to eliminate the culture media adhered to their roots. After this, we moved the cleaned plantlets to the adventitious shoot tray with the substrate for culture (peat soil, perlite, vermiculite, and coconut bran in a ratio of 3:1:1:1). In order to maintain relative humidity of 75–80%, we moved the plantlet tray to a greenhouse with a 16/8 light/dark cycle and covered it with plastic. The greenhouse had a light intensity of 42 mmol/m^2^/s and a temperature of 25 ± 3 °C. After two weeks, the plastic covers were progressively removed as the plantlets became accustomed to their potted surroundings. Depending on the growth of the regenerated plants, they were then placed into larger pots.

### 4.6. Genetic Homogeneity Analysis

For the purpose of evaluating the genetic fidelity, we chose 13 healthy regenerated plants at random along with the mother plant. Fresh leaves were rapidly frozen in liquid nitrogen and the genomic DNA of mango was extracted using cetyltrimethylammonium bromide (CTAB) technology [50]. The main steps for the extraction of DNA from mangoes using the CTAB method are as follows. To extract DNA from the mango leaves, 0.1 g of fresh leaves was finely ground into a powder. Subsequently, 700 µL of preheated (65 °C) CTAB extraction buffer was added to the powder, followed by gentle agitation. The mixture was then incubated in a 65 °C water bath for 1 h. Following incubation, 400 µL of chloroform/isoamyl alcohol (24:1) was added; it was mixed well, and centrifugation was performed at 12,000 rpm for 10 min, yielding a clear supernatant containing the DNA. Subsequently, anhydrous ethanol was added to the supernatant up to the 1.5 mL mark, and the mixture was frozen at −20 °C for 2 h to precipitate the DNA. It was then centrifuged at 12,000 rpm for 10 min in a centrifuge. Next, we discarded the supernatant, added 1 mL of 75% ethanol, mixed it well, and placed it in a centrifuge at 12,000 rpm for 1 min. Then, we immediately poured out the liquid and left the centrifuge tube to stand upright; when the ethanol had evaporated completely, we added 50 µL of deionized water. The quality and purity of the extracted DNA were subsequently assessed using a nucleic acid protein analyzer (Thermo Fisher Scientific, Waltham, MA, USA). Referring to the ISSR primers applied to mango in the published literature [51,52], ISSR primers with clear amplification and high polymorphism were ultimately selected (Table 2). The ISSR primers were synthesized by Thermo Fisher Scientific. We used a 20 µL amplified PCR system, and the components were as follows: 10 µL 2× SanTaq PCR Master Mix, 2 µL ISSR primers, 2 µL template DNA (200 ng/µL), and 6 µL ddH_2_O. The PCR amplification program was as follows: pre-denature at 95 °C for 4 min, denature at 95 °C for 40 s, anneal at the optimal annealing temperature (TM value of ISSR primer minus 1–3 °C) for 50 s, extend at 72 °C for 120 s for 40 cycles, finally extend at 72 °C for another 6 min, and store at 10 °C. The amplified products were separated on 1.2% agarose gel using 1× TAE (Tris acetate EDTA) buffer. Under UV light, the gel was recorded using a gel doc system (Bio-Rad, Hercules, CA, USA). The sizes of the amplicons were estimated through comparison with a 8 kb DNA marker (Vazyme, Nanjing, China). Following the evaluation of 20 ISSR primers that produced scoreable bands, repeatable bands were selected for further amplification.

### 4.7. Statistic and Data Analysis

The equations for the various in vitro regeneration parameters used in this study were as follows:

Adventitious shoot induction rate (%) = number of explants with adventitious shoots/number of initial explants × 100%;

Adventitious shoot elongation rate (%) = number of elongated shoots/number of shoots on elongation medium × 100%;

Root induction rate (%) = number of rooted plantlets/initial number of shoots × 100%.

Calculations were performed using Excel 2020 (Microsoft, Redomond, WA, USA). Statistical significance was calculated using one-way analysis of variance (ANOVA), and significant differences were defined using Duncan’s multiple comparison method with a significance level of *p* < 0.05 in SPSS 27 (IBM, Armonk NY, USA).

## 5. Conclusions

**Figure 8:**
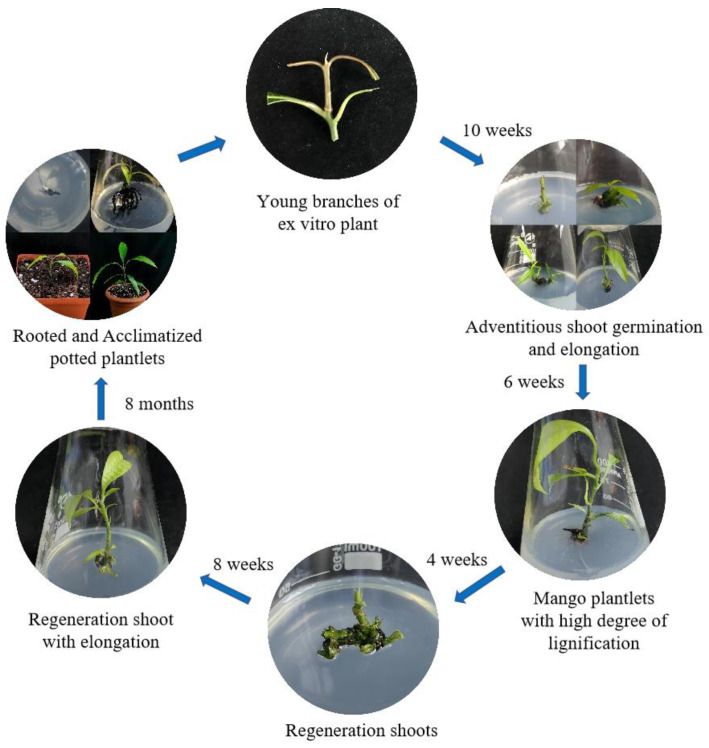
The flow scheme of an efficient shoot regeneration system for *Mangifera indica* L. Keitt. A protocol for efficient mango regeneration using branch explants was developed according to the above results.

In summary, we established an efficient shoot regeneration system with *Mangifera indica* L. (Figure 8). The induction of the adventitious shoots from sterile materials shows that a high degree of lignification can resolve the seasonal limitations of in vitro mango regeneration. Mango’s adventitious shoots are encouraged to form through the synergistic action of 6-BA and NAA. The optimal induction effect is obtained when the ratio of the two is 10/1. The most intensive formation of adventitious shoots was achieved with WPM. Mango’s adventitious shoot elongation is significantly affected by 6-BA, with 0.5 mg/L thought to be the suitable dosage. DCR with 3 mg/L IBA was the most suitable culture medium for the induction of roots. The ISSR analysis showed that there was no genetic fidelity difference between the regenerated plants and the mother plant of the system. We anticipate that this system may also be applicable to other mango varieties.

## Figures and Tables

**Table 1:** The effect of PGRs (in WPM) on adventitious shoot induction of *Mangifera indica* L. cv. Keitt.

6-BA (mg/L)	NAA (mg/L)	Adventitious Shoot Induction Rate (%)	Average Number of Adventitious Shoots per Explant
0.5	0.05	39.39 ± 0.48 ^f^	2.11 ± 0.32 ^e^
0.5	0.1	45.12 ± 1.27 ^e^	3.19 ± 0.60 ^d^
1	0.1	73.63 ± 0.55 ^a^	6.76 ± 0.64 ^a^
1	0.2	67.30 ± 0.83 ^b^	5.01 ± 0.58 ^b^
2	0.2	62.07 ± 1.11 ^c^	4.73 ± 0.60 ^c^
2	0.4	50.85 ± 1.61 ^d^	1.20 ± 0.52 ^f^

These values represent mean ± standard deviation. Duncan’s various lowercase letters show significant differences between the data at the 5% level, using the multiple range test at *p* < 0.05.

**Table 2:** The effect of basal medium on adventitious shoot induction of *Mangifera indica* L. cv. Keitt.

Basal Medium	Adventitious Shoot Induction Rate (%)	Number of Adventitious Shoots per Explant
MS	53.72 ± 0.89 ^c^	3.56 ± 0.73 ^c^
1/2 MS	56.70 ± 1.31 ^b^	4.83 ± 0.52 ^b^
WPM	63.87 ± 1.14 ^a^	5.21 ± 0.69 ^a^
DCR	47.92 ± 0.74 ^d^	2.15 ± 0.54 ^d^

These values represent mean ± standard deviation. Duncan’s various lowercase letters show significant differences between the data at the 5% level, using the multiple range test at *p* < 0.05. MS: Murashige and Skoog; 1/2 MS: half-strength Murashige and Skoog; WPM: wood plant medium; DCR: Douglas fir cotyledon revised.

**Table 3:** The effect of 6-BA concentration on adventitious shoot elongation of *Mangifera indica* L. cv. Keitt.

6-BA (mg/L)	Shoot Elongation (%)	Average Length (cm)	Description
0.1	75.00 ± 1.73 ^b^	4.51 ± 0.09 ^b^	Healthy green leaves
0.5	89.33 ± 2.51 ^a^	5.17 ± 0.16 ^a^	Healthy green leaves
1.0	66.00 ± 2.08 ^c^	4.26 ± 0.10 ^c^	Leaf edge coking

These values represent mean ± standard deviation. Duncan’s various lowercase letters show significant differences between the data at the 5% level using the multiple range test at *p* < 0.05.

**Table 4:** The effect of IBA concentration on rooting of adventitious shoots.

IBA (mg/L)	Mean Number of Roots per Explant	Adventitious Root Induction Rate (%)
1	1.33 ± 0.06 ^d^	33.03 ± 0.15 ^d^
2	4.23 ± 0.25 ^b^	49.23 ± 0.51 ^b^
3	6.43 ± 0.26 ^a^	66.13 ± 0.35 ^a^
4	2.60 ± 0.17 ^c^	38.63 ± 0.59 ^c^

These values represent mean ± standard deviation. Duncan’s various lowercase letters show significant differences between the data at the 5% level, using the multiple range test at *p* < 0.05.

**Table 5:** Detailed amplification results of ISSR primers used in the genetic stability of the in vitro-regenerated plantlets of *Mangifera indica* L. cv. Keitt.

Primer Code	Primer Sequence (5′–3′)	Annealing Temperature (°C)	No. of Scorable Bands	Size Range of Bands (bp)
UBC808	AGAGAGAGAGAGAGAGC	50	4	250–2000
UBC836	AGAGAGAGAGAGAGAGYA	48	9	250–2000
UBC848	CACACACACACACACARG	50	4	300–2000
UBC855	ACACACACACACACACYT	48	7	300–1200
UBC856	ACACACACACACACACYA	48	5	300–1800
UBC886	VDVCTCTCTCTCTCTCT	45	8	250–2000
UBC888	BDBCACACACACACACA	45	6	300–1500
UBC890	VHVGTGTGTGTGTGTGT	45	5	300–1200

## Data Availability

This published paper contains all of the data created and/or analyzed during this investigation.
